# Laboratory evaluation of the efficacy of lotilaner (Credelio™) against *Haemaphysalis longicornis* infestations of dogs

**DOI:** 10.1186/s13071-018-3032-0

**Published:** 2018-08-02

**Authors:** Hiroshi Otaki, Junko Sonobe, Martin Murphy, Daniela Cavalleri, Wolfgang Seewald, Jason Drake, Steve Nanchen

**Affiliations:** 1 Elanco Japan K. K., 4–15–1 Akasaka, Minato-ku, Tokyo, 107–0052 Japan; 2 Elanco Animal Health, Mattenstrasse 24a, CH-4058 Basel, Switzerland; 3 Elanco Animal Health, 2500 Innovation Way, Greenfield, IN 46140 USA

**Keywords:** Credelio, Dog, *Haemaphysalis longicornis*, Lotilaner, Ticks, Efficacy

## Abstract

**Background:**

Throughout Japan, Korea and China, *Haemaphysalis longicornis* ticks are vectors of *Babesia gibsoni*, which causes severe and progressive anemia in dogs. This study evaluated the efficacy of a single administration of lotilaner flavored chewable tablets (Credelio^TM^) against experimental canine *H. longicornis* infestations.

**Methods:**

Twenty-two healthy Beagles were ranked in descending order of counts of *H. longicornis* completed 48 h after challenge on Day -7. The 16 dogs with the highest live tick counts were blocked into pairs and within pairs randomized to either lotilaner-treatment at a minimum dose rate of 20 mg/kg or sham-treated controls. Treatment was administered within 30 ± 5 min following feeding on Day 0. Infestations with 50 unfed adult *H. longicornis* were completed on Days -2, 7, 14, 21, 28 and 35. Elizabethan collars were placed for 48 (± 2) h after each infestation and a T-shirt was placed on each dog to facilitate attachment. Ticks were counted *in situ* 12 and 24 h post-treatment and counted and removed after an additional 24 h (48 h after treatment) and 48 h after each post-treatment infestation. Dogs were sedated for tick challenges and counts. Live attached ticks on each dog were counted for efficacy assessments. Lotilaner was considered effective if the average tick attachment rate in the control group was at least 20%, if there was a statistically significant difference (*P* < 0.05) in mean tick counts between treated and control groups, and if the lotilaner-treated group had a calculated efficacy of at least 90%.

**Results:**

Average control group retention of the *H. longicornis* challenge exceeded 20% at each assessment. Lotilaner started killing *H. longicornis* ticks quickly, achieving 57.4% efficacy within 12 h. At 48 h post-treatment, and following each subsequent infestation, between-group differences in mean *H. longicornis* counts were significant (*P* < 0.0001). From 48 h post-treatment, through the final assessment on Day 37, lotilaner efficacy remained greater than 95%, including on Day 37 when efficacy was 98.4%.

**Conclusion:**

Lotilaner, administered to dogs orally at a minimum dose rate of 20 mg/kg is well tolerated, provides rapid reduction of existing *H. longicornis* tick infestations, and provides sustained residual protection for at least 35 days.

## Background

Lotilaner, the most recently approved isoxazoline, is presented in a flavored chewable tablet formulation (Credelio^TM^) and is indicated for the treatment of flea and tick infestations from dogs at the time of treatment and throughout the month following treatment. When administered orally to recently-fed dogs, lotilaner is rapidly absorbed with an onset of action of no less than 2 hours against fleas and 4 hours against the tick *Ixodes ricinus* [1–3]. Laboratory and field studies have shown that a single treatment can provide sustained activity for at least 1 month against fleas and a range of tick species including, but not limited to, *Rhipicephalus sanguineus*, *Amblyomma americanum*, *Dermacentor reticulatus*, *Dermacentor variabilis*, *Ixodes scapularis*, *I. hexagonus* and *I. ricinus* [3–7]. Over the month following treatment, lotilaner prevented pathogen transmission to dogs exposed to *D. reticulatus* ticks infected with *Babesia canis* [8].

The tick *Haemaphysalis longicornis* is prevalent in the Asia Pacific region, including in Australia, New Zealand and China, and is the most commonly found tick infesting dogs in Japan and Korea [9–12]. All life stages of this tick were also recently identified for the first time on a heavily infested sheep in the USA with no international travel history [13]. This tick is a vector of a range of pathogens that cause disease in mammals, and has been shown to transmit *Babesia gibsoni* to dogs, and to transmit agents of viral and rickettsial diseases to humans [12, 13]. In dogs, *B. gibsoni* infection presents with a range of progressive clinical manifestations that includes anemia, enlarged lymph nodes, diarrhea and elevated liver enzymes, and there has been a report of paraplegia [14, 15]. Prompt removal and sustained protection of dogs against this tick is therefore important, and there is a need for treatments that can provide immediate and sustained activity against *H. longicornis*. A study was undertaken with the objective of evaluating the efficacy of a single administration to dogs of lotilaner flavored chewable tablets at a minimum dose rate of 20 mg/kg, the label dose rate, against experimental challenges with unfed adult *H. longicornis* ticks.

## Methods

The study was designed largely in compliance with the World Association for the Advancement of Veterinary Parasitology (WAAVP) second edition guidelines for evaluating the efficacy of parasiticides for the treatment, prevention and control of flea and tick infestations of dogs and cats, with current local regulatory standards, and with the EMA Guideline for the testing and evaluation of the efficacy of antiparasitic substances for the treatment and prevention and control of tick and flea infestation in dogs and cats [16, 17]. All personnel involved in completing tick infestations and counts were blinded to treatment. Personnel who prepared and administered treatments did not participate in other study activities.

### Animals and housing

Twenty-two Beagle dogs, 11 males and 11 females, individually identified by ear tattoo, were available for inclusion in the study. A dog was eligible to be enrolled if on Day -7 it was clinically healthy, at least 7 months of age, weighed between 6.8 and 19.6 kg, and if it sustained a pre-treatment tick infestation of at least 10 live-attached ticks (20% of the challenge dose). Dogs were excluded if they had been included in any previous study or treated with an ectoparasiticide in the 60 days preceding selection. Dogs were individually housed in indoor cages. They were exercised daily in pairs from the same treatment group while staff cleaned cages, except for 48 (± 2) h between infestations and treatment administration when they were retained in their cages. Each cage contained rubber matting and wood shavings. Each dog was provided a toy. Dogs were fed a standard commercially available dry canine diet and water was provided *ad libitum*. Canned food was offered prior to treatment on Day 0 to ensure dosing under fed state. The general health of all dogs was observed by a trained technician at least once daily, except on Day 0 when clinical observations were completed by a veterinarian for each dog along with observations up to 4 h post-treatment.

### Tick infestations and counts

Tick infestations were completed with adult parthenogenetic *H. longicornis* ticks. These ticks had been bred at the study site (Shokukanken Inc., Maebashi-shi, Gunma, Japan) for two generations since being collected in Isezaki-shi, Gunma, Japan within the 12 months prior to beginning the study. Dogs were sedated with xylazine hydrochloride for tick challenge and counts. Infestations involved spreading 50 viable unfed ticks over the body on Days -7, -2, 7, 14, 21, 28 and 35, with focus on the ears, pinnae and face. The same technique was used for each dog. Elizabethan collars were placed on study dogs for 48 (± 2) h after each infestation, and a T-shirt was fixed on each dog to facilitate tick attachment. T-shirts remained in place until the tick count 48 h after infestation. Tick counting was performed by conducting a thorough whole-body examination beginning at the head and extending to the tail, and including the internal ears, pinnae and interdigital spaces. At the end of the whole-body examination, each dog was combed for 10 min, and if any tick was found during the final minute, combing was extended for an additional minute. On Days -5, 2, 9, 16, 23, 30 and 37 the ticks were counted and removed, 48 (± 2) h after treatment or after subsequent infestations. Additionally, at 12 (± 0.5) and 24 (± 1) h post-treatment, ticks were counted without combing, but were not removed. The numbers of live attached ticks on each dog were used for efficacy assessment calculations. Ticks were counted as “live” if showing any movement in response to a stimulus (exhaled air - CO_2_) and dead if showing no movement or reaction.

### Randomization and treatments

The 16 dogs (9 males and 7 females) with the highest tick counts that met all inclusion and none of the exclusion criteria, and had at least a 20% live tick attachment rate from a challenge on Day -7, were ranked in order of descending Day -5 tick count. Dogs were blocked into groups of two, one dog from each block was randomly assigned to either be treated with lotilaner or to be a sham-treated control, until all 16 were allocated. Randomizations (group assignments, pen assignments, tick counting order per time point) were performed using SAS® version 9.2.2.

Lotilaner was administered orally at as close as possible to the targeted minimum dose rate of 20 mg/kg. After administration of lotilaner, each animal was administered approximately 5 to 10 ml of water, *per os*, by syringe, to ensure and accelerate swallowing. All dogs consumed their full daily ration within approximately 30 min before dosing. Tablets were administered whole with each dose calculated based on body weights taken on Day -2. Control group dogs were sham-dosed on Day 0 using a process that matched the handling of the lotilaner-treated dogs, including removing the dog from its cage and administering water orally by syringe.

### Assessment of efficacy

Efficacy was determined by the reduction in live attached tick counts on lotilaner-treated dogs, relative to control dogs, at 12 h (Day 0), 24 h (Day 1) and 48 h after treatment, on Day 2 and after re-infestations on Days 9, 16, 23, 30 and 37. The experimental unit was the individual dog. Arithmetic and geometric mean group efficacies were calculated according to the formula:

$$ \mathrm{Efficacy}\kern0.5em \left(\%\right)\kern0.5em =\kern0.5em 100\kern0.5em \times \kern0.5em \left(\mathrm{Mc}\hbox{-} \mathrm{Mt}\right)\kern0.5em /\kern0.5em \mathrm{Mc} $$

where Mc is the mean number of live attached ticks on dogs in the untreated control group, and Mt is the mean number of live attached ticks on dogs in the treated group. The SAS procedure ‘Proc mixed’ was used for the comparison of treatment groups by analysis of variance; separate calculations were performed for each time point. Geometric means were calculated using log-transformed counts (count + 1) with one (1) subsequently subtracted from the result. All hypotheses were tested at a two-sided 0.05 level of significance. Lotilaner was considered effective if the average tick attachment rate in the control group was at least 20%, if there was a statistically significant difference (*P* < 0.05) in mean live tick counts between the treated group and the control group, and if the treated group had a calculated efficacy of at least 90%.

## Results

The dose rates of lotilaner administered to study dogs ranged from 20.13 to 24.35 mg/kg. There were no adverse events in either group. From each infesting dose of 50 *H. longicornis*, live attached tick counts in the control dogs ranged from 10 up to 24, with the average infestation remaining above 20% (range 27.3–39.0%) at each assessment, thereby meeting the requirement for adequate infestation and efficacy comparison of the treated group.

**Fig. 1:**
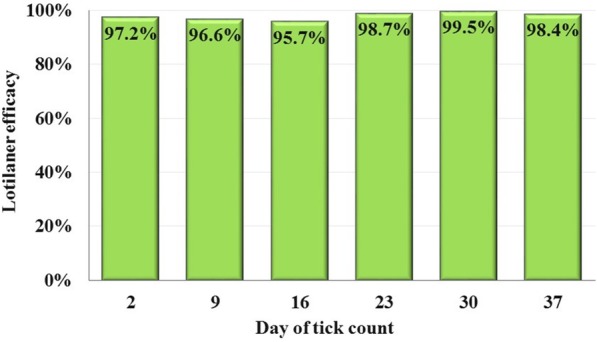
Percent reduction in geometric mean *Haemaphysalis longicornis* tick counts of lotilaner-treated dogs compared to untreated control group dogs

Within 48 h post-treatment, three lotilaner-group dogs were free of live attached ticks, and the remaining five dogs in this group each had a single live attached tick, resulting in an efficacy of 97.2% (Table 1). The maximum number of live attached ticks found on any lotilaner-group dog in the post-treatment period was two. On Day 9, there were two dogs with one tick and two dogs with two ticks. On Day 16, there were two dogs with one tick and three dogs with two ticks. Only two lotilaner-treated dogs had ticks on Day 23, one dog on Day 30 and the three dogs infested on Day 35 each had only a single tick at the Day 37 assessment. At 48 h post-treatment, and following each subsequent infestation, between-group differences in mean *H. longicornis* counts were significant (*P* < 0.0001). From 48 h post-treatment through the final assessment on Day 37 lotilaner efficacy remained greater than 95%, including on Day 37 when efficacy was 98.4% (Fig. 1). Efficacy based on geometric means at 12 h post-treatment was 57.4% (arithmetic mean efficacy 57.8%) and at 24 h was 89.5% (89.0%).

**Table 1:** Results of tick infestations based on live attached ticks 48 h post-treatment and following post-treatment infestations

		Day of tick count					
		2	9	16	23	30	37
Control	Arithmetic mean ± SD	19.4 ± 2.3	17.3 ± 5.1	18.8 ± 3.8	19.5 ± 4.6	17.1 ± 4.1	18.9 ± 2.7
	Geometric mean	19.3	16.6	18.4	19.0	16.7	18.7
Lotilaner	Arithmetic mean ± SD	0.6 ± 0.5	0.8 ± 0.9	1.0 ± 0.9	0.4 ± 0.7	0.1 ± 0.4	0.4 ± 0.5
	Efficacy (%)	96.8	95.7	94.7	98.1	99.3	98.0
	Geometric mean	0.5	0.6	0.8	0.3	0.1	0.3
	Efficacy (%)	97.2	96.6	95.7	98.7	99.5	98.4
Comparison		*t*_(14)_ = 19.3; *P*<0.0001	*t*_(14)_ = 11.7; *P*<0.0001	*t*_(14)_ = 12.1; *P*<0.0001	*t*_(14)_ = 15.9; *P*<0.0001	*t*_(14)_ = 22.9; *P*<0.0001	*t*_(14)_ = 20.0; *P*<0.0001

*Abbreviation*: *SD* standard deviation

## Discussion

Prior to the introduction of the isoxazolines there were no published reports of reliable efficacy of any compound against *H. longicornis.* In a study investigating the efficacy of topically applied formulations of imidacloprid/permethrin and fipronil/(S)-methoprene, ticks were applied to a shaved area on study dogs [18]. Conclusions about the efficacy of either product are limited because there were only three dogs in each group, there were no whole-body tick count assessments, no validation that limiting assessment to a small area of shaved skin is representative of the whole body, and no assessments from four days after treatment. That report therefore provides little information to indicate that either of those topically applied products would achieve an efficacy against *H. longicornis* that would approach what has been shown for afoxolaner in an earlier study [19], and what was demonstrated for lotilaner in our study.

In fact, the sustained efficacy of lotilaner against *H. longicornis* shown in our study compares favorably with the results of that afoxolaner study. Afoxolaner was shown to have an efficacy (based on geometric means) of 100% at 48 h post-treatment and at 48 h post-infestation on Day 9 [19]. Efficacy then progressively declined to 91.9% by Day 30. A potential limitation of that study is that on most assessment days at least one control group dog had low tick counts (for instance on Day 2, one control group dog had retained only four ticks; on Days 16 and 23 the lowest control group count was 6). Thus, the infestation was not as robust in that study, possibly because the ticks used for infestations might not have been as viable as those in our study, or the technique of tick challenge and recovery may have been less rigorous than ours in which dogs were sedated for challenge and count procedures. The reductions in afoxolaner efficacy against *H. longicornis* from Day 16 parallel reports of its declining efficacy but still meeting WAAVP guidelines with respect to efficacy against other tick species during the month following treatment [20–22]. Nonetheless, the studies on afoxolaner and lotilaner are valuable in demonstrating that treatments are now available that can help control infestations with *H. longicornis,* with efficacy continuing for a month following treatment. These results provide further evidence that lotilaner can be a valuable tool in helping to prevent the transmission of tick-borne pathogens to treated dogs.

## Conclusion

This study demonstrates that lotilaner given orally at a minimum dose rate of 20 mg/kg is well tolerated, provides a rapid reduction of existing *H. longicornis* tick infestations of dogs, and provides sustained residual protection for at least 35 days against post-treatment challenge. In this study, lotilaner started killing *H longicornis* ticks within 12 hours.

## Abbreviations

Mc: Mean number of live ticks on dogs in the untreated control group
Mt: Mean number of live ticks on dogs in the treated group
SD: Standard deviation
WAAVP: World Association for the Advancement of Veterinary Parasitology
